# A generalized population dynamics model for reproductive interference with absolute density dependence

**DOI:** 10.1038/s41598-017-02238-6

**Published:** 2017-05-17

**Authors:** Daisuke Kyogoku, Teiji Sota

**Affiliations:** 1 Department of Zoology, Graduate School of Science, Kyoto University, Kitashirakawa-Oiwake-cho, Sakyo, Kyoto 606-8502 Japan; 2 Faculty of Science and Technology, Ryukoku University, 1-5, Seta Oe-cho, Otsu, 520-2194 Japan

## Abstract

Interspecific mating interactions, or reproductive interference, can affect population dynamics, species distribution and abundance. Previous population dynamics models have assumed that the impact of frequency-dependent reproductive interference depends on the relative abundances of species. However, this assumption could be an oversimplification inappropriate for making quantitative predictions. Therefore, a more general model to forecast population dynamics in the presence of reproductive interference is required. Here we developed a population dynamics model to describe the absolute density dependence of reproductive interference, which appears likely when encounter rate between individuals is important. Our model (i) can produce diverse shapes of isoclines depending on parameter values and (ii) predicts weaker reproductive interference when absolute density is low. These novel characteristics can create conditions where coexistence is stable and independent from the initial conditions. We assessed the utility of our model in an empirical study using an experimental pair of seed beetle species, *Callosobruchus maculatus* and *Callosobruchus chinensis*. Reproductive interference became stronger with increasing total beetle density even when the frequencies of the two species were kept constant. Our model described the effects of absolute density and showed a better fit to the empirical data than the existing model overall.

## Introduction

Interspecific mating interactions are likely to occur between closely related species during secondary contact after allopatry owing to incomplete species recognition. The decreased fitness associated with interspecific mating interactions (termed reproductive interference) can influence the distribution and abundance of organisms through its effects on population dynamics^1, 2^. For example, reproductive interference was reported to have caused the displacement of native species by invasive congeners in various taxa^3–7^. Reproductive interference is thus a major concern in population management, particularly when invasive species are involved. Reproductive interference occurs as a result of individual interactions between males and females of different species, and the incidence of such interactions depends on the abundance of each species. Due to this density-dependent effect, a population dynamics model is required to forecast the demographic consequences of reproductive interference.

The existing population dynamics models of reproductive interference^8–10^ assume a specific behavioural mechanism: sterile interspecific fertilisation with a single-mating female the species recognition of which is error prone. In these models, the effects of reproductive interference on individual fitness are dependent solely upon the relative abundances of the interacting species, because the species identity of a female’s single mate determines her fitness; absolute density is irrelevant. However, several behavioural mechanisms of reproductive interference have been documented in animals^1, 2^, including persistent mating attempts^11^, interspecific copulation^12, 13^ and interspecific fertilisation^14–16^. These different behavioural mechanisms may ultimately have different effects on population dynamics. Particularly, absolute density and relative frequency are expected to affect the degree of fitness loss when the encounter rate of individuals is important (e.g., persistent mating attempts)^17^. Therefore, it would be useful to develop a general theoretical framework to predict population dynamics that is adaptable to various causal mechanisms.

Here, we aimed to develop a generalized population dynamics model with quantitative predictive power and also to empirically evaluate its applicability to an actual interspecific interaction. A pair of seed beetle species, *Callosobruchus maculatus* (Fabricius) and *Callosobruchus chinensis* (Linnaeus), provides an ideal model system for research on reproductive interference. Males of both species indiscriminately attempt to mate with females of the other species, even in the presence of their own conspecific females^18^. These promiscuous interspecific mating attempts result in near-unilateral interspecific copulation between *C. maculatus* females and *C. chinensis* males, repeated occurrence of which decreases female fecundity through physical injury to the reproductive tract^13, 19^. This reproductive interference by *C. chinensis* on *C. maculatus* has demographic consequences, with *C. maculatus* becoming extinct within several generations in the presence of *C. chinensis* under laboratory conditions^18, 20^.

To develop a general framework for population dynamics models of reproductive interference, we explicitly considered the process through which the abundances of interacting species affect the rate at which individual females interact with heterospecific males. We applied this framework to *Callosobruchus* seed beetles and derived an analytical population dynamics model for reproductive interference between them. We further performed a single-generation experiment using *C. maculatus* and *C. chinensis* to evaluate the practical applicability of the new model; the fecundity of *C. maculatus* was quantified in various densities of the two species. Note that our experiment was performed to determine the applicability of our model to actual biological systems rather than to predict the outcome of a specific competition experiment.

## The model

We modelled the secondary contact between closely related animal species where males indiscriminately try to mate with females of either species, and heterospecific females consequently incur fecundity loss without hybridisation. We assume that the cost to heterospecifically courting males has a negligible effect on the population dynamics of their own species, because the operational sex ratio is often male-biased^2^. In addition, we consider only reproductive interference by heterospecific males; although conspecific males can also affect female fitness^21^, we ignore this effect. Organisms are assumed to be polygamous and non-territorial. We did not consider interspecific interactions other than reproductive interference (e.g., resource competition^22, 23^), because they are beyond the scope of this paper.

We begin with a logistic differential equation for mathematical simplicity as in Kuno^9^ and Yoshimura and Clark^10^; note that Ribeiro and Spielman^8^ used a discrete generation model, to which our arguments presented below can be readily applied. We consider populations of males and females of species 1 and 2: *M*_1_, *F*_1_, *M*_2_ and *F*_2_. Let *s*_*i*_ denote the sex ratio (ratio of males) of species *i* (*i* = 1, 2), *D*_*i*_ the density independent mortality rate and *H*_*i*_ the coefficient of conspecific density dependence. Also, let *B*_*ij*_ denote the potential birth rate of species *i* when males of species *j* (*i* ≠ *j*) interfere with their reproduction. The dynamic equation for species *i* is then

1 $$\frac{d{F}_{i}}{dt}={F}_{i}[(1-{s}_{i}){B}_{ij}-{D}_{i}-{H}_{i}({F}_{i}+{M}_{i})]$$

2 $$\frac{d{M}_{i}}{dt}={F}_{i}{s}_{i}{B}_{ij}-{M}_{i}[{D}_{i}+{H}_{i}({M}_{i}+{F}_{i})].$$

By letting *M*_*i*_ + *F*_*i*_ = *N*_*i*_, equations (1) and (2) reduce to a single equation:

3 $$\frac{d{N}_{i}}{dt}={N}_{i}[(1-{s}_{i}){B}_{ij}-{D}_{i}-{H}_{i}{N}_{i}].$$

*B*_*ij*_ should depend on the incidence of interactions with males of species *j*, i.e.${B}_{ij}={B}_{ij}({I}_{ij})$, where *I*_*ij*_ is the average rate at which a female interacts (e.g., mates) with males of species *j*. *B*_*ij*_ is expected to be maximised when *I*_*ij*_ = 0 and to be decreased with increasing *I*_*ij*_, asymptotically approaching 0. As a simple approximation, we assume

4 $${B}_{ij}({I}_{ij})={B}_{i0}\cdot \exp (-{\alpha }_{ij}{I}_{ij}),$$

where *B*_*i*0_ is the intrinsic birth rate, and *α*_*ij*_ is the parameter controlling the fitness impact of interspecific interactions. *I*_*ij*_ should be affected by the abundances of both species 1 and 2^6, 18, 24^.

We assume that male mating attempts follow the type II functional response^25^. Suppose that a male of species *j* seeks mates for a time period *T* and encounters females of species *i* and *j* at rates *λ*_*ij*_ and *λ*_*jj*_, respectively; then the male attempts heterospecific mating at the rate

$$\frac{{\lambda }_{ij}T}{T+{\lambda }_{ij}T{h}_{ij}+{\lambda }_{jj}T{h}_{jj}}=\frac{{\lambda }_{ij}}{1+{\lambda }_{ij}{h}_{ij}+{\lambda }_{jj}{h}_{jj}},$$

where *h*_*ij*_ and *h*_*jj*_ are the “handling time”, or the time the male spends in a single encounter with a female of species *i* and *j*, respectively. Let us assume that *λ*_*ij*_ and *λ*_*jj*_ depend on female densities: ${\lambda }_{ij}={e}_{ij}(1-{s}_{i}){N}_{i}/S$ and ${\lambda }_{jj}={e}_{jj}(1-{s}_{j}){N}_{j}/S$, where *e*_*ij*_ and *e*_*jj*_) are female-species-specific mate-searching efficiency of the male and *S* is the area of the habitat. When male–male interference can be ignored, *s*_*j*_ *N*_*j*_ males of species *j* in area *S* collectively attempt heterospecific mating at a rate

$${s}_{j}{N}_{j}\cdot \frac{{e}_{ij}(1-{s}_{i}){N}_{i}/S}{1+{e}_{ij}{h}_{ij}(1-{s}_{i}){N}_{i}/S+{e}_{jj}{h}_{jj}(1-{s}_{j}){N}_{j}/S}.$$

These mating attempts are distributed over (1 − *s*_*i*_)*N*_*i*_ females; hence, on average, each female experiences mating attempts at a rate of

5 $$\begin{array}{c}\frac{1}{(1-{s}_{i}){N}_{i}}\cdot {s}_{j}{N}_{j}\cdot \frac{{e}_{ij}(1-{s}_{i}){N}_{i}/S}{1+{e}_{ij}{h}_{ij}(1-{s}_{i}){N}_{i}/S+{e}_{jj}{h}_{jj}(1-{s}_{j}){N}_{j}/S}\\ \,=\,\frac{{e}_{ij}{s}_{j}{N}_{j}/S}{1+{e}_{ij}{h}_{ij}(1-{s}_{i}){N}_{i}/S+{e}_{jj}{h}_{jj}(1-{s}_{j}){N}_{j}/S},\end{array}$$

which gives *I*_*ij*_ in equation (4). Thus, by equations (4) and (5), we obtain the birth rate of species *i*:

6 $${B}_{ij}({I}_{ij})={B}_{i0}\cdot \exp (-{\alpha }_{ij}\frac{{e}_{ij}{s}_{j}{N}_{j}/S}{1+{e}_{ij}{h}_{ij}(1-{s}_{i}){N}_{i}/S\,+\,{e}_{jj}{h}_{jj}(1-{s}_{j}){N}_{j}/S}).$$

By letting *α*_*ij*_ *e*_*ij*_ = *a*_*ij*_, *e*_*ij*_ *h*_*ij*_ = *b*_*ij*_ and *e*_*jj*_ *h*_*jj*_ = *b*_*ij*_, equations (3) and (6) gives

7 $$\frac{d{N}_{i}}{dt}={N}_{i}[(1-{s}_{i}){B}_{i0}\cdot \exp (-\frac{{a}_{ij}{s}_{j}{N}_{j}/S}{1+\{{b}_{ij}(1-{s}_{i}){N}_{i}+{b}_{jj}(1-{s}_{j}){N}_{j}\}/S})-{D}_{i}-{H}_{i}{N}_{i}].$$

Here, parameter *a*_*ij*_ represents the “interference efficiency” of heterospecific males, i.e., the combined effects of mate-searching efficiency (*e*_*ij*_) and fitness impact per encounter (*α*_*ij*_). Similarly, parameter *b*_*ij*_ and *b*_*jj*_ represent the “interference inefficiency” (i.e., how much time a male wastes in “handling” a female).

The first term within the brackets of equation (7) cannot be reduced to the function of the ratio of two species (*N*_*i*_/*N*_*j*_); both absolute density and species frequency affect the fitness impact of reproductive interference. Equation (7) predicts stronger reproductive interference at higher density even when the species frequency is constant (*B*_*ij*_(*N*_*i*_, *N*_*j*_) > *B*_*ij*_(*kN*_*i*_, *kN*_*j*_) if and only if *k* > 1). This property is qualitatively different from previous models such as Kuno’s^9^,which is equivalent to the following expression:

8 $$\frac{d{N}_{i}}{dt}={N}_{i}[(1-{s}_{i}){B}_{i0}\frac{{s}_{i}{N}_{i}}{{s}_{i}{N}_{i}+{i}_{ij}{s}_{j}{N}_{j}}-{D}_{i}-{H}_{i}{N}_{i}],$$

where *i*_*ij*_ is the coefficient of reproductive interference (see also refs 8, 10). In equation (8), the intensity of reproductive interference (the first term in the brackets) is dependent on species frequency but not on their absolute density. It should be noted that equation (5) and therefore equation (6) approximately becomes the function of the species ratio (*N*_*i*_/*N*_*j*_) when *N*_*i*_/*S* and *N*_*j*_/*S* are sufficiently large.

**Figure 1:**
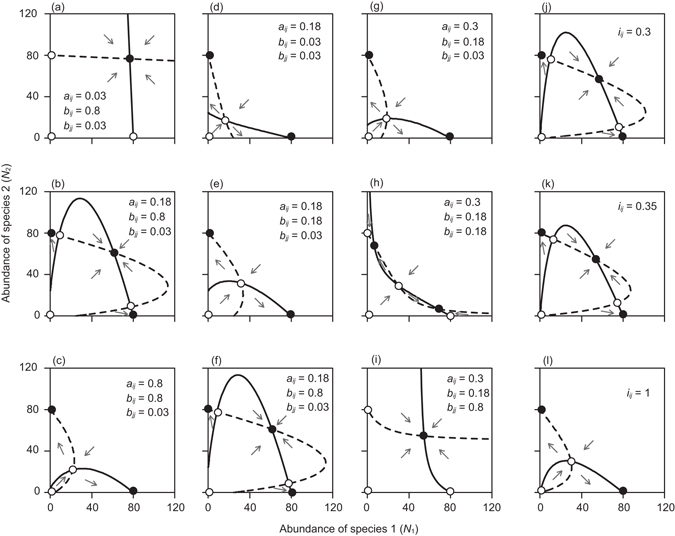
Parameter dependence of the zero-growth isocline for the generalized model (**a**–**i**) and the conventional model (**j**–**l**), with identical parameter values for the two species. Solid and broken lines are isoclines for species 1 and 2, respectively. Filled and open circles are stable and unstable equilibria, respectively. In (**a**–**c**, **d**–**f** and **g**–**i**) *a*_*ij*_, *b*_*ij*_ and *b*_*jj*_ in equation (7) are varied, respectively. In (**j**–**l**) *i*_*ij*_ in equation (8) is varied. Arrows indicate increase/decrease of *N*_1_ and *N*_2_ in each region of the phase plane. Other parameters used: (*B*_*i*0_, *s*_*i*_, *s*_*j*_, *S*, *D*_*i*_, *H*_*i*_) = (20, 0.5, 0.5, 1, 2, 0.1) for equation (7) and (*B*_*i*0_, *s*_*i*_, *s*_*j*_, *D*_*i*_, *H*_*i*_) = (20, 0.5, 0.5, 2, 0.1) for equation (8).

By solving *dN*_*i*_/*dt* = 0, we obtain the zero-growth isocline for species *i*, which is

9 $${b}_{jj}(1-{s}_{j}){N}_{j}=\frac{{a}_{ij}{s}_{j}\{{b}_{ij}(1-{s}_{i}){N}_{i}+S\}}{{a}_{ij}{s}_{j}+{b}_{jj}(1-{s}_{j})\mathrm{ln}\,\frac{{D}_{i}+{H}_{i}{N}_{i}}{(1-{s}_{i}){B}_{i0}}}-{b}_{ij}(1-{s}_{i}){N}_{i}-S,$$

for equation (7) and

10 $${i}_{ij}{s}_{j}{N}_{j}={s}_{i}{N}_{i}[\frac{(1-{s}_{i}){B}_{i0}}{{D}_{i}+{H}_{i}{N}_{i}}-1],$$

for equation (8). Figure 1 compares the isoclines of equations (7) and (8) assuming identical parameter values between the two species. Equation (7) produces unique and more diverse results than equation (8) depending on the parameter values. First, equation (7) always predicts positive population growth when the total density of the two species is sufficiently low (i.e., *N*_*i*_ + *N*_*j*_ ≈ 0) (Fig. 1a–i). This reflects the density dependence of reproductive interference; the isocline of equation (7) does not intersect the origin of the phase plane unless ln[(1 − *s*_*i*_)*B*_*i*0_/*D*_*i*_] = 0. Furthermore, when *a*_*ij*_/*b*_*ij*_ ≤ [(1 − *s*_*j*_)/*s*_*j*_]ln[(1 − *s*_*i*_)*B*_*i*0_/*D*_*i*_] is satisfied, the population of species *i* always increases when its density is sufficiently low regardless of species *j*’s abundance (Fig. 1a,i) (i.e., the isocline of species *i* does not intersect *N*_*j*_ axis). When *a*_*ij*_/*b*_*ij*_ > [(1 − *s*_*j*_)/*s*_*j*_]ln[(1 − *s*_*i*_)*B*_*i*0_/*D*_*i*_] is satisfied, the isocline of species *i* intersects *N*_*j*_ axis at *N*_*j*_ = [*S*/*b*_*jj*_(1 − *s*_*j*_)][*a*_*ij*_ *s*_*j*_/{*a*_*ij*_ *s*_*j*_ − *b*_*jj*_(1 − *s*_*j*_)ln[(1 − *s*_*i*_)*B*_*i*0_/*D*_*i*_]} −1]. Conversely, the isocline of equation (8) always intersects the origin in the phase plane due to the absence of density dependence of reproductive interference (Fig. 1j–l, see also refs 9, 10). Second, equation (7) can produce a single stable equilibrium, where two species coexist regardless of the initial abundances of the two species (Fig. 1a,i). In contrast, multiple equilibria always appear in equation (8), and a small population of the invading species cannot establish itself when the population of resident species is large (i.e., priority effect). Additionally, equation (7) can produce two stable equilibria where two species coexist (Fig. 1h). Note that the population densities of the two species at these equilibria can be highly asymmetric even when two species have identical parameters, a case where equation (8) produces a stable coexistence equilibrium with *N*_*i*_ = *N*_*j*_ (Fig. 1j,k).

The parameters controlling the intensity of reproductive interference in equation (7) are *a*_*ij*_, *b*_*ij*_ and *b*_*jj*_. These parameters have different effects on the model predictions. First, when the fitness impact of a single heterospecific male *a*_*ij*_ is large, the population growth of species *i* is reduced. This effect is especially important when species *j* is relatively more abundant than species *i* (Fig. 1a–c). Second, the dilution effect of increasing conspecific females *b*_*ij*_ has a positive effect on the population growth of species *i*, and its effect is more pronounced at intermediate *N*_*i*_, because the per capita reduction in fitness due to reproductive interference decreases with increasing conspecific density. The dilution effect of increasing heterospecific female abundance *b*_*jj*_ also has a positive effect on the population growth of species *i*. However, this effect is most pronounced when *N*_*i*_ is small because *b*_*jj*_ has larger scaling effect on equation (5) when *N*_*i*_ is small.

## Empirical study

### Data collection

We performed reaction surface analysis^26^, in which two species were combined at various abundances. We used the jC-F strain of *C. chinensis* ^27^ and the hQ strain of *C. maculatus* ^28^. Using the same combination of strains, Kishi *et al*.^18^ found that the fecundity of the hQ strain of *C. maculatus* was reduced by males of the jC-F strain of *C. chinensis*, but not by *C. maculatus* males. Therefore, our model’s assumption of no conspecific male effect was likely to be satisfied for this combination of strains. Equal numbers of *C. maculatus* males and females were introduced into Petri dishes (90-mm in diameter × 20 mm high) containing 20 g of adzuki beans, *Vigna angularis* (Willd.) ‘Dainagon,’ on which females laid eggs during their lifespan of about a week. We simultaneously introduced *C. chinensis* males into the dish. We used only virgin beetles collected within 24 h of emergence. Among experimental groups, the number of *C. maculatus* pairs ranged from 1 to 10, and the number of *C. chinensis* males ranged from 0 to 10 individuals. These density ranges were chosen because the conspecific density dependence effect, that is not distinguished from reproductive interference effect, would be negligible at such low density levels^29^. A total of 110 different combinations of *C. chinensis* and *C. maculatus* densities were prepared, each with three replications. When there were no hatched eggs in the dish, we prepared a fourth replicate. After beetles’ death, we counted the number of eggs in each dish, which is hereafter referred to as fecundity. This experiment was performed under laboratory conditions (30 °C, RH 70%, 16 L:8D).

### Model fitting and statistical analyses

Using our experimental results, we compared model fit to the data between the new and conventional models based on the Akaike Information Criterion (AIC) and determined statistical significance of the model fitting using likelihood ratio test (LRT)^30^. To fit the models to the data of seed beetles, which have discrete generations, we used difference equations analogous to equations (7) and (8), respectively:

11 $${N}_{i,t+1}={N}_{i,t}[\frac{1}{2}{R}_{i}\cdot \exp (-\frac{{a}_{ij}{N}_{j,t}/S}{1+{b}_{ij}{N}_{i,t}/2S})-{H}_{i}{N}_{i,t}]$$

and

12 $${N}_{i,t+1}={N}_{i}[\frac{1}{2}{R}_{i}\frac{{N}_{i,t}}{{N}_{i,t}+2{i}_{ij}{N}_{j,t}}-{H}_{i}{N}_{i,t}].$$

Note that *s*_*i*_ = 0.5 (for *C. maculatus*) and *s*_*j*_ = 1 (for *C. chinensis*) in our experiment and *b*_*jj*_ in equation (7) cannot be estimated. Here, *R*_*i*_ represents the maximum individual female fecundity and *N*_*i*,*t*_ represents the population of species *i* at generation *t*. We fitted the models to our experimental results by maximum likelihood estimation assuming a Gaussian distribution for total fecundity. The variance in total fecundity was assumed to be proportional to female abundance (see also Supplementary information). We observed a slight increase in fecundity over the course of the experiment (1,166 days) for unknown reasons, and therefore incorporated the date that a given experiment was conducted into the model (*R*_*i*_ was assumed to be the function of the date *d*; *R*_*i*_ = *R*_*i*0_ + *c*_*i*_ *d*, where *R*_*i*0_ is initial per capita fecundity and *c*_*i*_ is the coefficient controlling the effect of the date). We found that total fecundity was zero in five replications (mostly in treatments with a single ovipositing female), and we performed model selection by both omitting and including these zero-fecundity data. *S* in equation (7) was set to 1. All statistical analyses were performed using R software version 2.15.1^31^.

## Results

**Figure 2:**
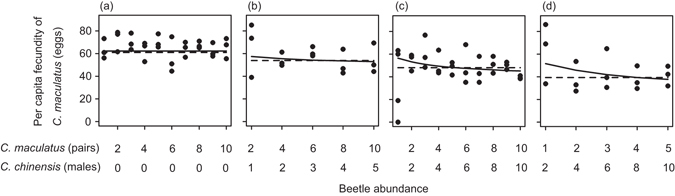
Comparison of experimental data and predictions of the fitted model at different frequencies of *C. maculatus* and *C. chinensis*. In each panel, per capita fecundity of *C. maculatus*, including zero-fecundity data, is plotted against total beetle density with identical *C. maculatus*: *C. chinensis* ratio; 1:0 in (**a**), 2:1 in (**b**), 1:1 in (**c**) and 1:2 in (**d**). The solid line represents the prediction of the generalized model (equation (11) with *H* = 0). The broken line represents the prediction of the conventional model (equation (12) with *H* = 0). For graphical purposes, we show per capita fecundity and not total fecundity. Estimated parameters: (*R*_0_, *a*, *b*, *c*) = (58.3, 0.119, 0.275, 0.0162) for equation (11) and (*R*_0_, *i*, *c*) = (57.4, 0.272, 0.0156) for equation (12). The date was set to the median of dates (day 230) in drawing model predictions. Note that the scales of the horizontal axes are not the same between panels.

We confirmed the absence of significant conspecific density dependence of individual fecundity using our data (Fig. 2a, Supplementary Table S1). Yet, in the presence of *C. chinensis*, individual fecundity of *C. maculatus* tended to decrease with increasing total density of the two species with constant *C. maculatus*: *C. chinensis* ratios (Fig. 2b–d). Furthermore, this density dependence was more pronounced when *C. chinensis* ratio was higher (Fig. 2). This absolute density dependence was captured by our model, but not by the conventional model (Fig. 2). When we fitted the models ignoring conspecific density dependence (i.e. *H*_*i*_ = 0 in equations (11) and (12)), our model (equation (11)) was the better in terms of AIC regardless of including or excluding the zero-fecundity data. In the analysis including zero-fecundity data (*n* = 335), the AIC of equation (11) was 3,423.1, which was lower than the AIC = 3,442.7 for equation (12), indicating the better fit of equation (11). This difference in AIC corresponds to a significant difference in the descriptive power of the models (LRT: *F*_1,330_ = 22.0, *P* < 0.0001). Estimated parameters are reported in the legend of Fig. 2. All results remained almost qualitatively and quantitatively unchanged when zero-fecundity data were omitted (*n* = 330) (Supplementary Table S2). Additionally, these results were unaffected by conspecific density dependence (Supporting information).

## Discussion

Our population dynamics model, which incorporates both the effects of absolute density and frequency dependence of reproductive interference, uniquely predicts stronger reproductive interference at higher absolute density even when *N*_*i*_:*N*_*j*_ ratio is the same. This absolute density dependence of reproductive interference yields diverse predictions depending on the parameters (Fig. 1). When applied to empirical data, our model appropriately described the absolute density dependence of reproductive interference (Fig. 2) and showed a slightly better fit to the data. These results suggest that caution is required when assuming reproductive interference to be purely frequency-dependent, especially in quantitative prediction of population dynamics. Although the observed difference in descriptive power was relatively small when equations (11) and (12) were applied to the empirical data, this may have been due to our experimental design with relatively narrow density regime, which we chose so that the conspecific density dependence of fecundity could be ignored. Larger difference in descriptive power may be observed in experiments in wider density regimes or in other organisms.

The parameter *a*_*ij*_ is the product of *α*_*ij*_ and *e*_*ij*_, *b*_*ij*_ of *e*_*ij*_ and *h*_*ij*_ and *b*_*jj*_ of *e*_*jj*_ and *h*_*jj*_. Explicit consideration of these parameter compositions enables biological interpretation of the parameter dependence of model predictions. First, it should be noted that *a*_*ij*_ and *b*_*ij*_ are not entirely independent, as they both include *e*_*ij*_ (e.g., male mobility) as a component. Interestingly, *e*_*ij*_ has contradictory effects on the intensity of reproductive interference. On the one hand, higher *e*_*ij*_ leads to a stronger interfering effect per single heterospecific male via its effect on *a*_*ij*_. On the other hand, higher *e*_*ij*_ enhances the dilution effect per single female through its effect on *b*_*ij*_. Overall, however, larger *e*_*ij*_ strengthens reproductive interference by narrowing the area of positive population growth on the phase plane (Fig. 1c,e), because *e*_*ij*_ has a larger scaling effect on the numerator than on the denominator of the exponential function in equation (7). Second, *α*_*ij*_ controls only the interfering effect of a single heterospecific male, which is not surprising from its definition (equation (4)). Third, *h*_*ij*_ scales the dilution effect per female through its effect on *b*_*ij*_. When interspecific copulation occurs, for example, *h*_*ij*_ is affected by its duration. Copulation duration varies markedly across taxa. For example, mating in the guppy (*Poecilia reticulata*) takes only about 1 s^32^, while that in the milkweed leaf beetle (*Labidomera clivicollis*) lasts up to 42 h^33^. Our model predicts that, with other factors being equal, a reproductively interacting species pair with larger *h*_*ij*_ (e.g., long interspecific copulation) is more likely to stably coexist (Fig. 1d–f), and *h*_*jj*_ will have similar effects. However, this result follows our assumption that heterospecific mating does not interfere with conspecific insemination. This assumption was applicable to our experimental setting, but may not be true in all cases. Finally, we note that relative values of *b*_*ij*_ and *b*_*jj*_ may vary across systems. For example, when males prefer conspecific to heterospecific females^17^, males may pursue conspecific females more persistently, resulting in *h*_*ij*_ < *h*_*jj*_. Alternatively, conspecific females may evolve counter adaptations against persistent male courtship which would allow them to more readily escape from male mating attempts than heterospecific females^34^ (resulting in *h*_*ij*_ > *h*_*jj*_).

Our model predicts positive population growth at sufficiently low total density of the two species (Fig. 1a–i). This reflects our assumption that the fitness loss of a female depends on the encounter rate with heterospecific males, which is expected to be low when the males are sparsely distributed. Weak reproductive interference at low densities may be common in nature, where organisms are likely to be distributed more sparsely than in laboratory environments such as Petri dishes. In a Swedish lake, the native crayfish *Astacus astacus* has declined in abundance, and reproductive interference by invasive crayfish *Pacifastacus leniusculus* appears to have contributed to the decline^3^. That decline began when the two species became abundant, having initially had low population densities^3^. Relatively strong reproductive interference at higher population densities may have caused the delayed decline of *A. astacus*.

We assumed type II functional response in formulating interspecific interaction rate (equation (5)). We made this assumption to describe reproductive interference in *Callosobruchus* seed beetles. This assumption may require modification depending on the reproductive biology of the organisms considered. For example, the existence of learning behaviours may violate our assumption. In some damselfly species, females have colour dimorphism and a male changes his preference for female morphs depending on his past experience^35^. In such species, a male may similarly change his strictness of female species discrimination depending on past experience. Female learning behaviour may also alter the incidence of costly mating interactions with heterospecific individuals. The lack of conspecific exposure is known to weaken premating reproductive isolation between species^4, 36^. For example, the ranid frog *Rana latastei* shows a drastic decline in reproductive success when housed with large numbers of *Rana dalmatina* males^4^. Hettyey and Pearman^4^ suggested that this strong reproductive interference in an extreme social environment may be due to the breakdown of species recognition by females. These plastic changes in behaviour will alter the probability at which interspecific encounters translate into costly mating behaviour and may thereby alter population dynamics (e.g. compare refs 37 and 38).

We ignored at least two factors when developing the model that can be included into the model to describe various systems. First, conspecific as well as heterospecific males may affect population dynamics^39^. Males have evolved harmful reproductive traits that are costly to females in many taxa^21, 40^, including *Callosobruchus* seed beetles^41, 42^. We ignored the effects of conspecific males, because they have much smaller effect on the fitness of *C. maculatus* females than heterospecific (*C. chinensis*) males^18^. However, males of two species of true bugs, *Neacoryphus bicrucis* and *Margus obscurator*, comparably reduce the fecundity of females of the former species^43^. Second, we ignored interspecific interactions other than reproductive interference. It is necessary to incorporate appropriate modifications, if, for example, interspecific competition over shared resources occurs^22, 23^. Reproductive interference and resource competition are known to synergistically impede species coexistence^10, 44^. Therefore, it is obvious that our isocline analysis (Fig. 1) overestimates the likelihood of coexistence as no interspecific resource competition was assumed. Because we explicitly considered the process through which heterospecific males reduce female fecundity when developing the model, these factors can be readily included by modifying the model.

Our model for interspecific reproductive interference may also be applied to conspecific male–female interactions for species in which males affect female fitness (sexual conflict^21^). For example, our model provides theoretical support for frequency-dependent selection in the damselfly *Ischnura senegalensis*, in which females have colour dimorphism^45^. At the first mating on a given day, males of this species mate with a female without female morph preference; this first mating is the basis for a learned preference for a female morph thereafter on that day. The probability that a male will form a preference for either female morph depends solely on the frequency of the morph in the population. Thus, a larger proportion of males in a population develop a preference for the dominant female morph, which produces negative frequency-dependent selection favouring the less abundant morph^45^. Here, for dominant females to incur more frequent mating attempts, the per capita rate of receiving mating attempts must depend on the absolute densities of harassing males and harassed females (but not on their relative abundance, which should be identical between morphs). Our models may similarly be applied to predict the demographic consequences of harmful male behaviour^39, 46^.

Despite the merits of our generalized model in describing reproductive interference, conventional models may still be appropriate in some cases. Equations (7) and (11) were designed to describe fecundity reduction that is dependent on the rate of interspecific encounters. Reproductive interference in *Callosobruchus* seed beetles occurs via interspecific copulation, repeated occurrence of which results in injury to the female reproductive tract^13, 19^; thus, interspecific encounter rate is crucial. On the other hand, conventional models were developed primarily assuming sterile interspecific mating of monandrous females^8–10^. When this assumption is met, the conventional models are expected to be more appropriate than our model. Therefore, our study highlights the importance of understanding the mechanisms of reproductive interference in predicting its effect on population dynamics.

In conclusion, our generalized model can flexibly describe various conditions, and is a useful tool for quantitatively predicting the dynamics of a population affected by reproductive interference. As we explicitly considered the process through which behavioural interactions affect individual fitness, the model can be readily modified and applied to various systems. Further studies are required to examine the versatility of our model and absolute density dependence of reproductive interference in other systems.

## Electronic supplementary material

Supplementary info
