# Supplementary material for: A generalized population dynamics model for reproductive interference with absolute density dependence
Source: Sci Rep. 2017 May 17;7:1996. doi: 10.1038/s41598-017-02238-6 (PMC5435698; doi:10.1038/s41598-017-02238-6)
Supplement: Supplementary file 1 — Supplementary info [file 41598_2017_2238_MOESM1_ESM.pdf]

A generalized population dynamics model for reproductive interference with absolute density dependence

Daisuke Kyogoku[*, 1, 2], Teiji Sota[1]

[1]Department of Zoology, Graduate School of Science, Kyoto University

[2]Present affiliation: Faculty of Science and Technology, Ryukoku University.

*Author for correspondence

Supplementary Methods. Statistical test for conspecific density dependence of *C. maculatus*.

We fitted a statistical model to the subset of our empirical data consisting of replications without *C. chinensis* males (1–10 *C. maculatus* pairs, n = 30) to examine the conspecific density dependence of per capita fecundity in *C. maculatus*. We assumed a Gaussian distribution for per capita fecundity. When mean per capita fecundity y is affected by conspecific density, it can be described as

$$y = \beta_0 - \beta_1 N_t, \qquad \text{(S1)}$$

where β_0 is the average per capita fecundity without density dependence, and β_1 is the coefficient of the density dependence. We also observed fecundity increase during the course of the experiment, which requires an additional term in equation (S1). Thus, mean total fecundity in a replicate can be de described as

$$y(1-s)N_t = (1-s)N_t\left[\beta_0 - \beta_1 N_t + \beta_2 d\right]. \qquad \text{(S2)}$$

Here, d is the date on which the replicate was prepared, and β_2 represents the effect of the date. Probability theory says that the sum of n independent identically distributed Gaussian variables with variance σ^2 follows the Gaussian distribution whose variance is $n\sigma^2$. Hence we assumed a Gaussian distribution whose mean was $y(1 - s_i)N_t$ and whose variance was proportional to $(1 - s)N_\mathrm{t}$. We fitted this model using the maximum likelihood method (Supplementary Table S1).

Supplementary Table S1. Estimated parameters of equation (S2).

Variable	Estimate (95% confidential interval)
β_0	64.32 (57.65, 70.99)
β_1	0.308 (−0.137, 0.753)
β_2	0.012 (0.007, 0.017)

Supplementary Table S2. Estimated parameters of equations (11) and (12) fitted to data without zero-fecundity.

Model	R_0	(a, b) or i	c	AIC*
Equation (11)	58.9	(0.107, 0.227)	0.0157	3,301.2
Equation (12)	57.7	0.268	0.0151	3,335.3

*The difference in AIC between the models is significant ($F_{1,325}$ = 37.6, P < 0.0001)

Supplementary Data. Model fitting with conspecific density dependence.

We reported model (equations (11) and (12)) fitting without conspecific density dependence in the main text. We similarly fitted the models with conspecific density dependence to the same data. When applied to data that included zero-fecundity (n = 335), the AIC of equation (11) was 3,423.4, which was smaller than the AIC = 3,427.3 for equation (12), indicating the better fit of equation (11). This difference in AIC corresponds to a significant difference in the descriptive power of the models (LRT: $F_{1,329}$ = 5.77, P = 0.02). Estimated parameters were (R, a, b, c, H) = (61.0, 0.152, 0.428, 0.0161, 0.0935) for equation (11) and (R, i, c, H) = (65.6, 0.277, 0.0158, 0.236) for equation (12). All results remained almost qualitatively and quantitatively unchanged when zero-fecundity data were omitted. When applied to data without zero-fecundity (n = 330), the AIC of equation (11) was 3,301.1, which was smaller than the AIC = 3,312.5 for equation (12), again indicating the better fit of equation (11). This difference in AIC corresponds to a significant difference in the descriptive power of the models (LRT: $F_{1,324}$ = 13.4, P = 0.0003). Estimated parameters were (R, a, b, c, H) = (61.6, 0.134, 0.352, 0.0156, 0.0930) for equation (11) and (R, i, c, H) = (66.0, 0.272, 0.0154, 0.242) for equation (12).
